# Relationships between Plasma Pyrophosphate, Vascular Calcification and Clinical Severity in Patients Affected by Pseudoxanthoma Elasticum

**DOI:** 10.3390/jcm11092588

**Published:** 2022-05-05

**Authors:** Georges Leftheriotis, Nastassia Navasiolava, Laetitia Clotaire, Christophe Duranton, Olivier Le Saux, Saïd Bendahhou, Audrey Laurain, Isabelle Rubera, Ludovic Martin

**Affiliations:** 1 University Hospital Nice, Vascular Physiology and Medicine Unit, 06000 Nice, France; 2 Université Côte d’Azur, LP2M, UMR CNRS 7370, LabEx ICST, 06107 Nice, France; laetitia.clotaire@etu.univ-cotedazur.fr (L.C.); christophe.duranton@univ-cotedazur.fr (C.D.); said.bendahhou@univ-cotedazur.fr (S.B.); laurain.a@chu-nice.fr (A.L.); isabelle.rubera@univ-cotedazur.fr (I.R.); 3 PXE Reference Center, MAGEC Nord, University Hospital of Angers, 49000 Angers, France; nastassia.navasiolava@chu-angers.fr (N.N.); lumartin@chu-angers.fr (L.M.); 4 Department of Cell and Molecular Biology, John A. Burns School of Medicine, University of Hawaii at Manoa, Honolulu, HI 96817, USA; lesaux@hawaii.edu

**Keywords:** pseudoxanthoma elasticum, inorganic pyrophosphate, ectopic calcification, cardiovascular, PHENODEX score, disease severity, PXE natural history

## Abstract

Pseudoxanthoma elasticum (PXE; OMIM 264800) is an autosomal recessive metabolic disorder characterized by progressive calcification in the skin, the Bruch’s membrane, and the vasculature. Calcification in PXE results from a low level of circulating pyrophosphate (PPi) caused by ABCC6 deficiency. In this study, we used a cohort of 107 PXE patients to determine the pathophysiological relationship between plasma PPi, coronary calcification (CAC), lower limbs arterial calcification (LLAC), and disease severity. Overall, our data showed a deficit in plasma PPi in PXE patients compared to controls. Remarkably, affected females showed higher PPi levels than males, but a lower LLAC. There was a strong correlation between age and PPi in PXE patients (r = 0.423, *p* < 0.0001) but not in controls (r = 0.059, *p* = 0.828). A weak correlation was found between PPi and CAC (r = 0.266, *p* < 0.02); however, there was no statistically significant connection with LLAC (r = 0.068, *p* = 0.518) or a severity score (r = 0.077, *p* = 0.429). Surprisingly, we found no significant correlation between plasma alkaline phosphatase activity and PPi (r = 0.113, *p* = 0.252) or between a 10-year cardiovascular risk score and all other variables. Multivariate analysis confirmed that LLAC and CAC were strongly dependent on age, but not on PPi. Our data showed that arterial calcification is only weakly linked to circulating PPi levels and that time (i.e., age) appears to be the major determinant of disease severity and calcification in PXE. These data are important to better understand the natural history of this disease but also for the follow-up and management of patients, and the design of future clinical trials. Our results also show that PPi is not a good biomarker for the evaluation of disease severity and progression.

## 1. Introduction

Pseudoxanthoma elasticum (PXE; OMIM 264800), is an autosomal recessive metabolic inherited disorder characterized by progressive calcification in the skin, the Bruch’s membrane, and the vasculature [1,2,3]. PXE results from mutations in the *ABCC6* gene which encodes an ABC transporter primarily found in hepatic and renal tissues. Peripheral arterial disease is one of the major causes of morbidity in PXE [4,5] and a higher risk for premature coronary disease has also been reported both in patients [6] and heterozygous carriers [7]. In 2013, ABCC6 function was linked to the cellular efflux of ATP and subsequent generation of PPi [8].

Thus, ABCC6 deficiency leads to a significant deficit in circulating pyrophosphate (PPi), a major inhibitor of soft tissue calcification [9].

Up to now, the connection between calcification susceptibility and reduced plasma PPi has essentially been studied in KO mice for ABCC6 [10,11,12,13,14]. These knockout animals develop spontaneous mineralization in the vasculature, ocular, and renal tissues, as well as in the capsule of vibrissae [15], which serves as a marker for disease progression. Recently, Shimada et al. reported that a low plasma PPi level does not correlate with the calcification phenotype in various ABCC6-deficient mice or human patients, with identical ABCC6 disease variants developing either the relatively mild PXE symptoms or the severe generalized arterial calcification of infancy (GACI) [1]. This suggests that environmental factors and/or modifier genes participate in the development of ectopic calcification in PXE which could be independent of PPi level. Furthermore, as a link between PPi plasma levels and ectopic calcification is important for management patient and the future clinical trials, PPi has been considered as a biomarker in PXE, GACI, and other calcifying diseases.

In the present study, we examined the correlation between arterial calcification, disease severity, and PPi level in a cohort of PXE patients.

## 2. Materials and Methods

Medical records from 232 PXE patients from the French-based PXE reference center cohort localized at the University Hospital of Angers were analyzed retrospectively. One hundred and seven PXE patients were selected from this cohort based on biological, radiological, and clinical data. All PXE patients were subjected to a comprehensive screening program including dermatological (including skin biopsy), ophthalmological, and vascular examinations every 1–3 years by trained clinicians. The diagnosis of PXE was based on the revised criteria by Plomp et al. [2], and the phenotype characteristics were scored according to the organ system and severity PHENODEX scoring system [3]. The 10-year cardiovascular risk score for the French population was determined using the HeartScore website calculator (https://heartscore.escardio.org/2016/quickcalculator.aspx?model=EuropeLow, accessed on 1 January 2021). The renal function was determined using the MDRD-4 equation.

### 2.1. Ethical Committee Approval

The study was approved by the French ethical committee (reference DC 2011-1467 and AC-2014-2329) and ClinicalTrials.gov, accessed on 1 January 2022, #NCT01446380.

### 2.2. Vascular Calcification Scoring

Vascular calcification from coronary and lower limb arteries was determined from a noncontrast CT scan with a 64-slice MSCT scanner (Brilliance 64; Philips Healthcare, Hong Kong, China or Somaton Definition-Flash Radioc; Siemens, Singapore). Cardiac CT angiography was performed using a collimation of 40 × 0.625 mm and a rotation time of 0.4 s. The tube current was adjusted to body weight (range 240–400 mA) and 120 kV. For the quantitative assessment of coronary artery calcification (CAC), the Agatston score was calculated (4), using a 2.5 mm CT slice thickness and a detection threshold ≥130 HU involving ≥1 mm^2^ area/lesion. Lower limb arterial calcification (LLAC) scoring was performed on the volume/density data normalized to limb length (arterial tree of each lower limb was divided into aorto–iliac, femoro–popliteal, and below-knee segments). The LLAC score (expressed in HU/mm) was calculated as the sum of all segmental scores of both legs and normalized to the arterial lengths. The interobserver reliability of the analysis has been reported elsewhere [5].

### 2.3. Plasma Pyrophosphate

PPi quantification in ultrafiltrated patient plasma was performed using a modified method using ATP-sulfurylase (New England Biolabs, Ipswich, MA, USA, MO394L) in the presence of APS (adenosine-5′-phosphosulfate, Sigma-Aldrich, Burlington, MA, USA, A5508) as previously described [16,17]. Values for plasmatic PPi concentration in a non-PXE population (*n* = 26, control cohort) showed a median value of 1.53 µmol/L (irq = 0.43 µmol/L) and a mean value of 1.63 ±0.07 µmol/L ±SEM).

### 2.4. Plasma Alkaline Phosphatase (ALP) Activity

Plasma ALP activity was assessed using paranitrophenol at alkaline pH and absorbance at 450 nm according to standard clinical protocols.

### 2.5. Statistical Analyses

Data are presented as mean (±SD) or median (interquartile: iqr) according to the distribution of the variables. Statistical univariate correlations between the variables were determined using the Pearson or Spearman correlation tests and multivariate analyses were performed on selected variables. Data were analyzed using Stata 12.0 software (StataCorp, Texas, TX, USA). A *p* value <0.05 was considered significant, unless specified.

## 3. Results

### 3.1. PXE Patients Population

Data from the studied cohort are shown in Table 1. Over sixty-seven percent of the cohort of our study were females (72/107). The average age of this population is around 48.4 ±15.7 (Table 1). The mean 10-year cardiovascular risk in the overall population was 1% (iqr1).

Plasma PPi levels were measured twice in a subgroup of seven patients, including six females, within a three-year interval.

### 3.2. Assessment of PPi in Men and Women in PXE Patients

Female patients showed higher PPi and Pi, a lower 10-year cardiovascular risk score, and significantly lower LLAC scores as compared to PXE men. However, CAC values did show differences between genders but trended down for PXE women. No significant differences in the PPi/Pi ratio, ALP, glomerular filtration, and PHENODEX score were found between men and women.

As expected, the median (irq) plasma PPi was significantly lower in PXE patients (0.77 µmol/L (0.37 µmol/L)) than in healthy controls (1.53 µmol/L (0.43 µmol/L), *p* < 0.0001). Sixty-three percent of these samples (41 women, *p* < 0.05) had PPi levels lower than the mean value measured in controls (see Table 2). Compared to the PXE with a within-normal range PPi, PXE patients with a low PPi were mostly women and of younger ages, with lower Pi and PPi/Pi ratio (Table 2).

We found that plasma PPi increased in all seven patients with repeated measurements (mean interval = 2.4 years, range 1 to 3 years), from an average of 0.82 µmol/L (0.21 µmol/L) to 1.14 µmol/L (0.34 µmol/L, *p* = 0.018). Despite this increase, there was no significant change in Pi (1.06 mmol/L (0.27 µmol/L) vs. 1.09 mmol/L ((0.19 µmol/L), *p* = 0.735) in their PHENODEX score (8 (2) vs. 8 (1), *p* = 0.317) and ALP activity (68 ±6 UI/L vs. 68 ±7 UI/L, r = 0.868).

### 3.3. Relationships between PPi Level, Calcification Scores, and Disease Severity

**Figure 1:**
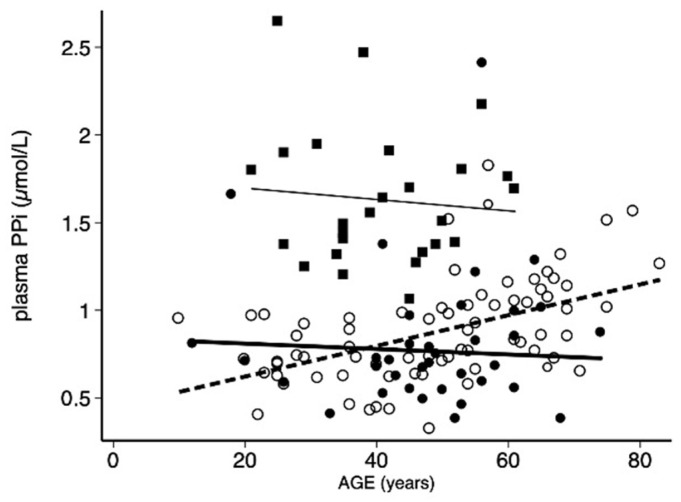
Distribution of plasma pyrophosphate (PPi) measured in controls (*n* = 25, squares) and PXE patients (circles): women (open circles) and men (filled circles) as a function of age (year).

Univariate analysis showed that plasma PPi correlated with age (r = 0.423, *p* < 0.0001, Figure 1), CAC (r = 0.493, *p* < 0.0001), LLAC (r = 0.570, *p* < 0.001), and the PHENODEX score (r = 0.500, *p* < 0.0001), but not with ALP (r = 0.121, *p* < 0.217). Remarkably, the correlation between PPi level and age was only significant in females (r = 0.560, *p* < 0.0001).

A weak, but still significant, relationship was found between PPi and CAC (r = 0.266, *p* < 0.02), though there was neither correlation with LLAC (r = 0.068, *p* = 0.518) nor with the PHENODEX score (r = 0.077, *p* = 0.429). The PHENODEX score strongly correlated with CAC (r = 0.422, *p* < 0.0001) and LLAC (r = 0.583, *p* < 0.0001). Surprisingly, there was no significant relationship between ALP and PPi concentration (r = 0.113, *p* = 0.252) as well as between the 10-year cardiovascular risk score and other variables. No significant association was found between PPi and age in healthy controls (r = 0.059, *p* = 0.828, Figure 1).

### 3.4. Statin-Treated PXE Patients

21 patients (14 females) were taking statins at the time of the study (*p >* 0.05 versus men, Table 3). Patients treated with statins were significantly older and exhibited higher CAC, LLAC scores, and PHENODEX score (Table 3).

**Figure 2:**
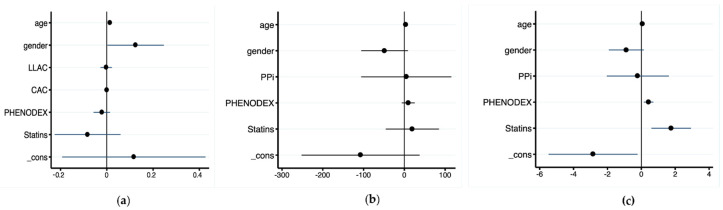
Coefficient plots from multivariable stepwise regression models with PPi (**a**), CAC (**b**) and LLAC (**c**) as dependent variables.

After multivariate stepwise analysis, only the associations between plasma PPi, age, and gender remained (Figure 2a). Multivariate stepwise analysis including CAC or LLAC as dependent variables and age, gender, PHENODEX, and statins as independent variables showed that CAC and age were linked (Figure 2b), whereas LLAC correlated significantly with age, PHENODEX, and statin (Figure 2c).

## 4. Discussion

This is the first study addressing actual correlation between plasma PPi, arterial calcification (in two different vascular beds), and a disease severity score (PHENODEX) in a large cohort (>100) of PXE patients. These patients had low levels of plasma PPi, a major calcification inhibitor produced by the liver and kidneys which regulates ectopic calcification in soft connective tissues.

### 4.1. Plama PPi Level in PXE

Based on our methodology for PPi measurement, we found that the average plasma level was 50% lower in PXE patients than in control individuals, which is consistent with previous findings reported on PXE patients [9,18]. Furthermore, the plasma PPi level was higher in female than in male PXE patients, but there was no statistical gender-related difference in the control groups. Interestingly, we found a strong positive correlation between PPi and age in female PXE patients, which was not observed in male PXE patients and healthy controls of both sexes. A similar progression in PPi levels with time was also observed in the subgroup of seven PXE patients who had two consecutive PPi measurements.

A recent study of a pediatric cohort of 200 children who underwent blood testing for medical conditions not affecting plasma PPi levels revealed no significant difference between genders and only a decreasing trend (not statistically significant) between age 0 to 18 [19]. To our knowledge, the only reported increase in PPi levels with age was in the urine of ABCC6^−/−^ mice [20]. In addition, we found in our cohort age-dependent and gender differences in plasma Pi, as previously observed in the general population [21,22], but there was no significant difference in the PPi/Pi ratio.

The reason for plasma PPi increase with age in our PXE cohort is unknown and somewhat counter intuitive, as a previous report showed an increase in calcification and symptoms severity with age [23]. A first explanation could be related to the level of ALP, the circulating fragment of membrane-bound enzymes that degrades PPi into Pi. ABCC6 mutant cells exhibit increased expression and activity of tissue-nonspecific alkaline phosphatase (TNAP), a major inducer of calcification, and the administration of TNAP inhibitor attenuates both the development and progression of calcification in ABCC6^−/−^ mice [24]. A decrease in ALP activity with age could explain higher levels of plasma PPi. A previous study involving a smaller number of PXE patients (*n* = 18) reported a weak negative association between PPi and TNAP in both PXE and controls [18]. However, due to the small size of the population, this correlation was not significant when PXE patients were considered alone. Similarly, in chronic kidney disease, low PPi levels were strongly associated with high levels of circulating ALP activity [16]. The same was observed during pregnancy in healthy females and both conditions could explain a decreased plasma PPi [25]. However, in our study, the lack of correlation between PPi and ALP suggests that this enzyme (and possibly TNAP, the tissue-bound ALP) does not significantly influence plasma PPi levels or the other factors are involved. For example, lower renal elimination of PPi could be a contributor. Although we couldn’t measure urine PPi, a progressive reduction in the renal filtration can be ruled out by the lack of a correlation between PPi and GFR. Interestingly, in the study of Bouderlique et al., the progressive increase in urine PPi in ABCC6^−/−^ mice suggested that PPi could be freely filtered with age [20]. Finally, the reason why PPi changed solely in female PXE patients over time remains unknown. Of note, on average there are twice as many female PXE patients, including in our cohort, and this skewed gender ratio is still unexplained.

### 4.2. PPi and Arterial Calcification

PXE is characterized by premature ectopic calcification in the wall of peripheral arteries, affecting mostly elastic fibers. This is responsible for the peripheral arterial disease associated with PXE [5,26]. The incomplete correlation we found between PPi and arterial calcification is of concern since both variables are potential biomarkers for disease progression. Additional studies are needed to address this issue.

In the recent literature, there are numerous examples of studies reporting a lack of correlation between plasma PPi and calcification susceptibility in both humans and animals. Inhibition of the TNAP attenuated ectopic mineralization in the ABCC6^−/−^ mice model without affecting PPi level [27]. Another study showed that, despite identical plasma PPi levels, ABCC6 knockout mice C57BL/6J showed much stronger susceptibility to mineralization than C3H/H1J mice with a naturally occurring inactivating ABCC6 mutation [1]. By contrast, Zhao et al. [28] showed that in the restoration of normal PPi levels in ABCC6^−/−^ mice expressing a human ENPP1 transgene, mineralization still developed independently of PPi plasma level. Two separate studies by Pomozi et al. showed a restoration of mineralization inhibition in humanized ABCC6^−/−^ mice without significant changes in plasma PPi [10,12]. Finally, the severe GACI phenotype of the milder PXE can result from mutation in either ABCC6 or ENPP1 and vastly different PPi levels. Indeed, ENPP1 is the bottleneck enzyme for PPi generation. In our study, the LLAC and CAC scores were lower in female patients than in males. One could easily be tempted to explain this finding by a higher plasma PPi level which also correlated with a lower cardiovascular risk. However, the multivariate analysis showed no significant link between PPi, LLAC, and CAC. However, the association between LLAC and the PHENODEX score suggested that LLAC could be a better biomarker of disease progression than CAC. Additionally, this is consistent with our previous studies comparing arterial calcifications in PXE and the general population [22].

Interestingly, an increased level of calcification in the lower limb arteries was strongly associated with statin use and that was independent of PPi levels. This was not the case for coronary arteries. Furthermore, observational studies in the general population have reported that statins differentially affect the progression of arterial calcification in coronary arteries vs. the aorta [29,30]. Atorvastatin has also been reported to slow the progression of calcification in the ABCC6^−/−^ murine model [31]. However, contrary to these previous studies, we found that statins had a strong pro-calcifying effect on the lower limb vasculature in PXE, but not in the coronaries. In light of a recent publication showing pro-atherogenesis and dyslipidemia effects associated with ABCC6 deficiency in both ABCC6^−/−^ mice and PXE patients [32], our data bring into question the cost/benefit ratio for the use of these drugs in PXE.

### 4.3. Duration of Exposure to A Low Plasma Level of PPi Is A Major Determinant of Arterial Calcification and Clinical Severity in PXE

An important finding of the study was the strong correlation between age, arterial calcification, and disease severity, which was independent of plasma PPi. The age-dependency of severity has been reported in another cohort, without any contribution of gender or genotype [23]. This finding suggests that age (i.e., the time of exposure to low PPi levels) remains a major determinant in the PXE phenotype. The strong impact of age underscores the progressive and cumulative nature of arterial calcification, similar to what is observed in the general population [33], but developing faster. Thus, we hypothesize that accumulation of calcification in the arterial wall could occur at a different rate depending on two main variables: the PPi deficit and time of exposure. This is well-illustrated in GACI patients where the extremely low plasma levels of PPi lead to acute calcification leading to a rapid demise of the patients [34,35]. By contrast, in PXE, the modest and chronic deficit of plasma PPi (about 50% lower than normal) causes calcification and clinical manifestations to develop over decades, as opposed to only weeks or months in GACI. Moreover, ectonucleotidic metabolic sources in peripheral tissues likely preserve a residual PPi production in PXE, thus limiting the impact of the PPi deficit [9]. Therefore, the design of future clinical trial endpoints and prognoses should take in consideration the duration of treatment as a function of age combined with the severity of the PPi deficit.

### 4.4. Study Limitations

This retrospective analysis was a transversal study of a subgroup of PXE patients issued from a large cohort.

Comparison with other studies were somewhat difficult since PPi plasma level is strongly dependent of the accuracy of measurement. However, the small longitudinal analysis performed in a subgroup of patient provided a clue to natural history of PPi within a limited three-year interval period.

Although our data confirm the differential impact of the low plasmatic PPi concentration on the vasculature as previously reported [22], our interpretation of the differences between CAC and LLAC was also limited by the lack of a control group. In the present study, analysis of the ectopic calcification process was limited to two arterial beds, whereas mineralization affects, and other connective tissues, such as the skin, the Bruch’s membrane, and renal parenchyma [11], which were not examined here. Furthermore, it has become clear that plasma PPi does not necessarily reflect local tissue production [9,36], which likely plays a non-negligible role in the phenotypic variability of PXE and which cannot be reliably determined at present.

## 5. Conclusions

Our study shows, for the first time, a significant role of age on plasma PPi levels in PXE, independently of AC and disease severity, and mainly in females patients. Although plasma PPi level may provide a biological clue for the clinical diagnosis, our data shed more light on the complexity of PPi homeostasis and its role on calcification and disease severity. This highlights the importance of taking these factors into account in the design of clinical endpoints, and also the need to increase the plasma level of PPi at an early stage in the disease. Finally, we strongly suggest that the benefice impact of statins in PXE should be re-examined.

## Figures and Tables

**Table 1:** Summary of biometric and biological variables. Comparisons between men and women PXE patients is shown. Data with a normal distribution are expressed as mean (±SD), others are expressed as median (interquartiles). PPi: inorganic pyrophosphate; Pi inorganic phosphate; ALP: alkalin phosphatase; GFR: glomerular filtration rate; CAC: Coronary arterial calcification; LLAC: Lower limb arterial calcification; CVR: 10 years—Cardiovascular Risk score.

	Men, *n* = 35		Women, *n* = 72		All, *n* = 107		*p* Value
**Age (years)**	47.7	±3.8	48.7	±16.7	48.4	±15.7	0.7542
**Body mass index (kg/m^2^)**	24.91	(3.55)	23.03	(5.35)	23.81	(5.14)	0.0756
**Total cholesterol (mmol/L)**	5.1	(1.3)	5.0	(1.5)	5.0	(1.3)	0.3729
**HDL cholesterol (mmol/L)**	1.3	(0.6)	1.4	(0.5)	1.4	(0.4)	0.0039
**GFR (mL/min/1.73 m^2^)**	115.5	±21.0	114.0	24.5	114.4	23.4	0.7589
**PPi (µmol/L)**	0.71	(0.31)	0.84	(0.33)	0.77	(0.37)	0.0299
**Pi (mmol/L)**	1.07	±0.21	1.17	±0.15	1.14	±0.18	0.0049
**PPi/Pi ratio**	0.66	(0.28)	0.73	(0.27)	0.7	(0.28)	0.3751
**ALP (UI/L)**	66	(22)	62	(23)	63	(24)	0.0649
**CAC (HU)**	36.3	(129)	2	(90)	7.5	(90)	0.0684
**LLAC (HU/mm)**	0.89	(2.76)	0.17	(1.88)	0.35	(1.95)	0.0452
**PHENODEX score**	5.9	±1.9	6.1	±1.9	6.0	±1.9	0.5846
**CVR (%)**	1	(0)	1	(1)	1	(1)	0.0500

**Table 2:** Statistical comparisons between PXE patients exhibiting a significantly lower or a nonsignificantly lower plasmatic PPi concentration (low or normal PPi) according to our laboratory reference control value for PPi. Data with a normal distribution are expressed as mean (±SD), others are expressed as median (interquartiles). PPi: inorganic pyrophosphate; Pi inorganic phosphate; ALP: alkaline phosphatase; GFR: glomerular filtration rate; CAC: Coronary arterial calcification; LLAC: Lower limb arterial calcification; CVR: 10 years—Cardiovascular Risk score.

	Normal PPi, *n* = 39		Low PPi, *n* = 68		All, *n* = 107		*p* Value
**Age (years)**	57	(18)	46.5	(18)	50	(22)	0.0006
**Gender (W/M)**	34/9		43/28				0.0500
**Body mass index (kg/m^2^)**	22.13	(5.60)	24.49	(4.06)	23.81	(5.14)	0.0675
**Total cholesterol (mmol/L)**	5.2	(0.9)	4.8	(1.4)	5.0	(1.3)	0.7998
**HDL cholesterol (mmol/L)**	1.5	(0.8)	1.4	(0.5)	1.4	(0.4)	0.0820
**PPi (µmol/L)**	1.05	(0.25)	0.69	(0.19)	0.77	(0.37)	9.2 × 10^−18^
**Pi (mmol/L)**	1.22	(0.13)	1.08	(0.25)	1.15	(0.24)	0.0009
**PPi/Pi ratio**	0.90	(0.25)	0.61	(0.16)	0.70	(0.28)	4.6 × 10^−15^
**ALP (UI/L)**	62	(29)	64	(20)	63	(24)	0.4754
**GFR (mL/min/1.73 m^2^)**	106.7	±23.6	118.9	±22.2	114.4	±23.4	0.0097
**CAC (HU)**	26.9	(150)	5.6	(46.2)	7.5	(90)	0.0872
**LLAC (HU/mm)**	0.66	(3.16)	0.32	(1.84)	0.35	(1.95)	0.5732
**PHENODEX score**	6.1	±1.9	6.0	±1.8	6.0	±1.9	0.8385
**CVR (%)**	1	(1)	1	(0)	1	(1)	0.5828

**Table 3:** Comparison between statine-treated and -untreated PXE patients. Data with a normal distribution are expressed as mean (±SD), others are expressed as median (interquartile). PPi: inorganic pyrophosphate; Pi: inorganic phosphate; ALP: alkalin phosphatase; GFR: glomerular filtration rate; CAC: Coronary arterial calcification; LLAC: Lower limb arterial calcification; CVR: 10 years—Cardiovascular Risk score.

	No Statine, *n* = 86		Statine, *n* = 21		All, *n* = 107		*p* Value
**Age (years)**	45.9	±15.8	58.5	±11.0	48.4	±15.7	0.0008
**Body mass index (kg/m^2^)**	23.53	(5.06)	24.91	(4.03)	23.81	(5.14)	0.1404
**Total cholesterol (mmol/L)**	5.2	(1.4)	4.7	(1.0)	5.0	(1.3)	0.1115
**HDL cholesterol (mmol/L)**	1.4	(0.4)	1.4	(0.5)	1.4	(0.4)	0.4138
**PPi (µmol/L)**	0.77	(0.36)	0.80	(0.33)	0.77	(0.37)	0.4102
**Pi (mmol/L)**	1.14	±0.18	1.12	±0.18	1.14	±0.18	0.5921
**PPi/Pi ratio**	0.69	(0.29)	0.71	(0.25)	0.70	(0.28)	0.3252
**ALP (UI/L)**	62	(25)	64	(22)	63	(24)	0.9999
**CAC (HU)**	1	(49)	56.9	(112)	7.5	(90)	0.0037
**LLAC (HU/mm)**	0.19	(1.43)	1.90	(7.47)	0.35	(1.95)	0.0004
**PHENODEX score**	6	(3)	7	(3)	6	(3)	0.0062
**CVR (%)**	1	(1)	1	(0)	1	(1)	0.4050

## Data Availability

The datasets generated during and/or analyzed during the current study are available from the corresponding author on reasonable request. Biological samples for the study are stored and available in the Angers’ biobank under the reference BB-0033-00038.
